# Associations between Body Segment Mass and Punch, Front Kick, or Countermovement Jump Performance in Military Cadets

**DOI:** 10.3390/sports12080205

**Published:** 2024-07-28

**Authors:** Michal Vagner, Jan Malecek, Vladan Olah, Petr Stastny

**Affiliations:** 1 Department of Sports Games, Faculty of Physical Education and Sport, Charles University in Prague, 162 52 Prague, Czech Republic; stastny@ftvs.cuni.cz; 2 Department of Military, Faculty of Physical Education and Sport, Charles University in Prague, 162 52 Prague, Czech Republic; jan.malecek@ftvs.cuni.cz (J.M.); olah@ftvs.cuni.cz (V.O.)

**Keywords:** martial art, close combat, biomechanics, body mass, dynamic forces

## Abstract

Despite the recognized influence of body mass on combat techniques, the relationship between body segment mass (BSM) and combat moves remains unexplored. This study aimed to examine the relationship between the striking arm mass (SAM), kicking leg mass (KLM), and body mass (BM) and the dynamic forces of direct punch (DP), palm strike (PS), elbow strike (ES), front kick (FK), and countermovement jump (CMJ) performance. Sixteen male military cadets (22.3 ± 1.8 years, 181.4 ± 7.0 cm, 82.1 ± 8.5 kg) performed combat techniques, with their performance measured by using a force plate and their body segment mass assessed by dual-energy X-ray absorptiometry. Spearman’s correlation analysis, the Wilcoxon test, and Cohen’s *d* were applied. The results indicated the relationship between the KLM or BM and the FK impulse (*r* = 0.64, *p* = 0.01; *r* = 0.52, *p* = 0.04, respectively) and CMJ impact force (*r* = 0.80, *p* ≤ 0.01; *r* = 0.70, *p* ≤ 0.01, respectively). The FK peak and impact forces were moderately correlated with the CMJ jump height (*r* = 0.74, *p* ≤ 0.01; *r* = 0.77, *p* ≤ 0.01). Moreover, the FK peak force was significantly higher than that for DP, PS, and ES (*p* ≤ 0.01, *d* = 3.32; *p* ≤ 0.01, *d* = 1.6; and *p* = 0.013, *d* = 1.3, respectively). The highest relationship was found between the KLM and the FK impulse; however, the difference in variability explained by the KLM versus the body mass was only 12%. This suggests that knowledge of the BSM did not provide a significantly better estimate of the dynamic forces of the punches and FKs than the knowledge of the BM.

## 1. Introduction

The physical and tactical training of the military is designed to maximize operational performance capacity [1]. Military training also includes close combat, including martial arts and combat sports techniques. Combat sports or martial arts often emphasize the importance of physical strength, speed, and technique [2,3,4]. The efficiency of combat moves, such as punches, kicks, and jumps, is a matter of skill but can also be related to the athlete’s body composition [5,6,7,8]. Based on this knowledge, researchers have developed an interest in how the body composition and somatotype influence the efficacy of these movements [5,9,10]. It is also known that enhancing the striking force of combat sport athletes involves effectively utilizing their body mass; this enhancement can refine movement techniques and consequently boost combat efficiency [11]. The existing studies primarily focus on the differences in the subjects’ weight, body composition, and somatotype estimates. However, the relationship between the mass of the arm and lower limb segments and the dynamic forces of punches and kicks remains unclear.

Previous research has demonstrated that maximal strength training and acceleration strength training can enhance kicking and fighting ability [4,12,13,14], and a higher acceleration of the lower body limbs was associated with a higher performance level in combat sports [13,15,16,17,18,19,20]. The ability to transition quickly from the eccentric to the concentric phase of muscle action is especially vital in executing combat moves, kicks, and jumps [21]. However, to evaluate the level of strength capabilities, regular, accurate, and reliable monitoring and testing are essential [22]. Countermovement jump (CMJ) testing is one of the most valid and frequent protocols for quantifying lower limb acceleration strength [17]. Therefore, this study also investigated the relationship between the CMJ, the kicking leg mass, and the dynamic forces of FKs. 

Studies dealing with analyzing the effectiveness of punches and kicks are based on Newton’s laws of motion. According to Newton’s second law, force is directly proportional to mass and acceleration. In combat sports, this principle suggests the relationship between the mass and the dynamic forces of the strike. Therefore, previous studies have explored the dynamic forces of punches and kicks [23,24,25,26,27,28,29]. Additionally, some studies have demonstrated that dynamic forces like peak or impact force within the kicks partially depend on a subject’s mass [6,7,8,30]. Other studies also considered the effective mass, which can be used to reveal how much percent of body mass contributes to the resulting strike force [11,31]. These findings underscore the relevance of body mass in combat techniques. 

Therefore, the study design aimed to explore (1) the relationship between the striking arm mass and the dynamic forces of the punches, (2) the relationship between the kicking leg mass and the dynamic forces of the front kick, (3) the relationship between the kicking leg mass and the CMJ performance, and lastly (4) comparing whether these aforementioned relationships will be higher than the relationship between the dynamic forces and the total body mass. 

## 2. Materials and Methods

### 2.1. Study Design

This cross-sectional quantitative study addressed the question of the relationship between the body mass segments (striking arm and kicking leg) and the dynamic forces of punches, front kicks, and CMJ performance. This study complied with ethical research standards and received approval from the Faculty of Physical Education and Sport Ethics Committee, Charles University (No. 085/2022). All procedures were conducted following the Declaration of Helsinki.

### 2.2. Procedure

**Figure 1:**
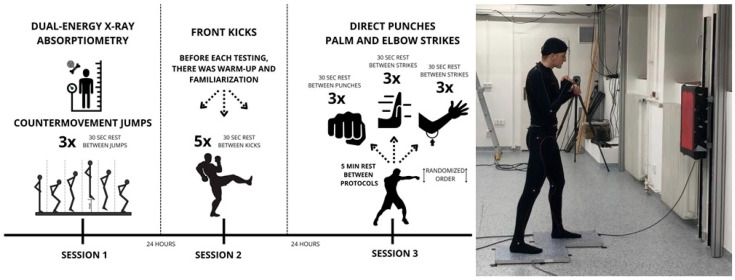
Testing protocol of this study and execution of the front kick and punches into a vertically positionable force plate.

The testing protocol of this study was compiled from three sessions (Figure 1). Session 1 began with measurements taken via dual-energy X-ray absorptiometry, followed by a series of countermovement jumps, in which participants performed three jumps with a 30 s rest interval between each jump. Session 2 was conducted 24 h after the first and focused on front kicks. The participants engaged in a warm-up and familiarization period before the test. Then, they executed five front kicks with a 30 s rest interval between each kick. Session 3, also held 24 h after the previous session, evaluated direct punches, palm strikes, and elbow strikes. The participants performed three sets of each type of strike with a 30 s rest interval between strikes. The session included a 5 min rest between different striking protocols, and the order of the strikes was randomized to prevent any order effect.

### 2.3. Participants

This study involved sixteen male military cadets (age: 22.3 ± 1.8 years, height: 181.4 ± 7.0 cm, mass: 82.1 ± 8.5 kg, all measurements reported as mean ± SD), selected based on their regular involvement in combat training and military physical training that commonly included punches, kicks, and jumps. The participants, all students from a military sports university, had been serving in the military for three years at the time of testing. None of these participants had any other experience with martial arts training apart from a regular previous three-year training in close army combat. The participants were familiar with the DP, PS, ES, FK, and CMJ experimental protocols. They had two regular lessons in close combat per week within their military practice during each semester. In two training sessions, the chosen techniques were practiced in detail to ensure consistent and accurate execution of each technique as per the study requirements. All the participants were informed about the study’s purpose and procedures and consented. All the participants were healthy and without traumatic injury affecting performance or musculoskeletal injuries occurring within three months before the start of this study.

### 2.4. Instruments

The methods and instruments used for measuring punches, front kicks, countermovement jumps, body mass, and body mass segments are listed in this section.

#### 2.4.1. Punches and Front Kicks

Following the 10 min warm-up, which included 5 min of relaxed-paced stationary cycling and dynamic exercises, such as calf raises, hip hinges, lunges, squats, and hopping, the participants underwent a familiarization process with the punches and kicks, encompassing five test strikes. They then adjusted their individual distances to the impact area. All the front punches and kicks began with a front-facing posture and were executed such that the hand made contact with the force plate at a height equivalent to the head and the foot contacted the plate at midsection height [12,29,32]. Following these preparatory activities, they were instructed to deliver each strike with maximum effort. A close-combat army instructor who holds a black belt in the Musado Military Combat System evaluated the correctness of the techniques for the kicks and punches. 

Each participant performed a series of combat moves, including three DPs, PSs, ESs, and five FKs (each strike or kick was followed by a 30 s rest period). The punches and FKs were executed into the force plate (Kistler 9281; Winterthur, Switzerland, adjusted at a minimum sampling rate of 1000 Hz) that was mounted in the front as the target. The height of the force plate was individualized for each participant. The force plate was covered by a mat (tatami 400 × 300 × 25 mm, StrongGear, Praha, Czech Republic) to reduce the risk of injury and connected to a computer with a 16-bit A/D board and BioWare V5.3.2.9 software. 

In the data processing phase, the recorded signals were first subjected to a high-pass Butterworth filter with a cutoff frequency of 5 Hz to remove low-frequency drifts and baseline noise. A zero-phase digital filtering technique was employed to prevent phase distortions. The peak force (N) was recorded, and the impact force (N) and impulse (N.s) were calculated using MATLAB software (version 1.8.0.121; MathWorks, Natick, MA, USA). The calculation involved extracting the maximum value of the 2 ms sliding mean net force over the contact period from all three axes (x, y, z) [12,33]. To ensure the reliability of these measures, a residual analysis was conducted to assess the consistency of the signal processing by examining the residuals of the fitted models across multiple trials. This analysis helped to validate the temporal stability and repeatability of the measurements.

#### 2.4.2. Countermovement Jump

At the lab, the participants started with a standardized warm-up, which included 5 min of relaxed-paced stationary cycling and dynamic exercises, such as calf raises, hip hinges, lunges, squats, and hopping. After this, they performed five less-intense CMJs, taking a 30 s rest between each [34]. After a warm-up and a brief 2 min rest, the participants, wearing the standard Czech military training footwear, positioned themselves on a calibrated force plate. During the performance of kicks, punches, and CMJ testing, the participants wore elastic suits from Qualisys (Figure 1). For the CMJ tests, they also wore standard Czech military training footwear. They were advised to remain stationary for 5 s and then leap as high as possible upon the research assistant’s signal. The CMJ routine consisted of a preliminary squat phase leading into a forceful jump, focusing on achieving maximum height and power. During these jumps, the participants kept their hands on their hips to focus on lower body strength, thereby limiting the involvement of the upper body. The objective was to land accurately back on the force plate, ensuring the entire jump motion was recorded [35]. Each person completed three maximum-effort CMJs, with the freedom to choose their countermovement depth and a 60 s rest between each jump [36]. The force plate was placed on the ground and connected to a computer with a 16-bit A/D board and BioWare V5.3.2.9 software. The maximum jump height (cm) and peak force (N) at the end of the breaking phase [34] were recorded, and the impact force (N) within the breaking phase was calculated as an impulse (N.s) from the initial contact to the time to reach the peak force [12] using MATLAB software (version 1.8.0.121; MathWorks, Natick, MA, USA). 

#### 2.4.3. Dual-Energy X-ray Absorptiometry

Upon morning arrival following an overnight fast, the participants prepared for a body composition analysis via dual-energy X-ray absorptiometry by removing all metallic and inorganic materials, including jewelry, belts, and any other accessories, to eliminate potential imaging artifacts. Following these initial preparations, the participants were instructed to lie motionless on the scanning table, adhering to standardized measurement conditions to ensure the accuracy and consistency of the dual-energy X-ray absorptiometry scans [37]. The assessment utilized a narrowed fan-beam dual-energy X-ray absorptiometry system (Lunar Prodigy; GE Healthcare, Madison, WI, USA), with the subsequent analysis performed using GE Encore 12.30 software to ensure precise body composition metrics. The dual-energy X-ray absorptiometry system‘s calibration was verified against a standard phantom to maintain consistent measurement accuracy [38]. For the purpose of this study, the whole-body mass, striking arm mass, and kicking leg mass were recorded.

### 2.5. Statistical Analyses

The values of the peak force (N), impulse (N·s), height (cm), concentric peak velocity (m/s), and impact force (N) were obtained from the force plates. The dual-energy X-ray absorptiometry scans measured the striking arm and the kicking leg (kg). The Wilcoxon test and Cohen’s *d* were used to compare the dynamic forces between the executed DPs, PSs, ESs (three strikes for each variable), and FKs (five front kicks). Significance was determined by the effect size using Cohen’s *d*, which can be interpreted as small (0.2 to 0.5), medium (0.5 to 0.8), and large (*d* > 0.8). 

The Spearman’s correlation coefficient was used to determine the relationships between the body segment mass and the performance metrics in the combat moves and the CMJ. The measured values of the punches and kicks were analyzed in both their original values and normalized values (punches normalized by the mass of the striking arm and front kicks normalized by the mass of the kicking leg) to account for individual differences in limb mass. The results for the dynamic forces were calculated as follows: for punches, the average value from three performed strikes was taken; for front kicks, the average from five performed kicks was used; and for the CMJ, the average from three performed jumps was considered for further analysis.

Statistical analyses were performed using SPSS version 25.0 (IBM) and Excel 2019 version 2312 (Microsoft, Redmond, WA, USA). A sensitivity analysis was conducted using G*Power (version 3.1.9.6) to determine the minimum detectable effect size of the Spearman’s correlation coefficient in our sample of 16 participants. Exact bivariate correlations were utilized with a two-tailed test, an alpha error probability set at 0.05, and a power (1-β) of 0.80 (80%). The computed sensitivity to detect significant correlations was *r* ≥ 0.64. Consequently, the strength of the correlations was interpreted as weak (<0.64), moderate (0.64–0.79), or strong (≥0.80). This approach ensured that our sample size was sufficient to detect a statistically significant correlation that would inform the impact of the body segment mass on the punches, kicks, and CMJ performance.

## 3. Results

Table 1 shows the descriptive statistics for the performance metrics of the direct punches, palm strikes, elbow strikes, front kicks, and countermovement jumps and the body segment mass assessed using dual-energy X-ray absorptiometry. The results indicate that the FKs achieved the highest values of all dynamic forces, and the PSs and ESs achieved higher values than the DPs in the peak and impact forces but not in the impulse. The Wilcoxon test revealed that the FK peak force was higher than that for the DP, PS, and ES (*p* ≤ 0.01, *d* = 3.32; *p* ≤ 0.01, *d* = 1.6; and *p* = 0.013, *d* = 1.3, respectively) as well as the FK impact force (*p* ≤ 0.01, *d* = 3.98; *p* ≤ 0.01, *d* = 1.89; and *p* = 0.01, *d* = 2.13, respectively). When comparing punches, the Wilcoxon test showed that the DP peak and impact forces were lower than those for the PS and ES (*p* ≤ 0.01, *d* = 1.93; *p* ≤ 0.01, *d* = 2.75; and *p* ≤ 0.01, *d* = 2.58; *p* ≤ 0.01, *d* = 2.62, respectively). 

### 3.1. Correlation Analysis between Body Segment Mass and Punches, Front Kicks, or CMJ

The Spearman’s correlation coefficient was used to determine the association between the body segment mass (including striking arm, kicking leg, and body mass) and the dynamic forces of the DPs, PSs, ESs, FKs, and CMJs (Table 2). A positive significant correlation was revealed between the impact force of the CMJ and the kicking leg mass and body mass. Moreover, a positive significant correlation was revealed between the impulse of the front kick and the kicking leg mass. However, a weak relationship existed between the peak and impact forces of the DP, PS, and ES and the striking arm mass. 

### 3.2. Correlation Analysis between Front Kick Dynamic Forces and CMJ Performance

Table 3 depicts the association between the dynamic forces of the front kick and CMJ, and between the normalized dynamic forces of the front kick and CMJ performance. The strongest positive correlation was revealed between the jump height of the CMJ and the front kick peak or impact force. However, when we used normalized values of front kick dynamic forces, the correlation with the jump height of the CMJ decreased. Furthermore, another association was revealed between the peak velocity and the front kick peak force. Moreover, when using normalized values of the front kick peak force, the association with the peak velocity of the CMJ remained almost the same. Finally, interestingly, the impact force of the CMJ had weak associations with the front kick peak and impact force. 

### 3.3. Graphical Representation of Findings

**Figure 2:**
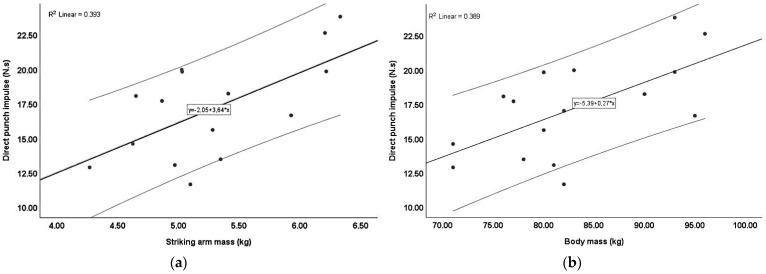
Correlation between the impulse of the direct punch and striking leg mass or body mass: (**a**) striking arm mass and direct punch impulse; (**b**) body mass and direct punch impulse.

Several key findings emerged from the correlation analysis. Figure 2 illustrates the correlation between the striking arm mass and the impulse generated during the direct punch (Figure 2a) and between the body mass and the impulse generated during the direct punch (Figure 2b). The regression line suggests a positive relationship, indicating that the impulse generated in a direct punch tends to increase as the striking arm mass or body mass increases. However, the relationship between the striking arm mass and the direct punch impulse was nearly identical to that between the body mass and the direct punch impulse. 

**Figure 3:**
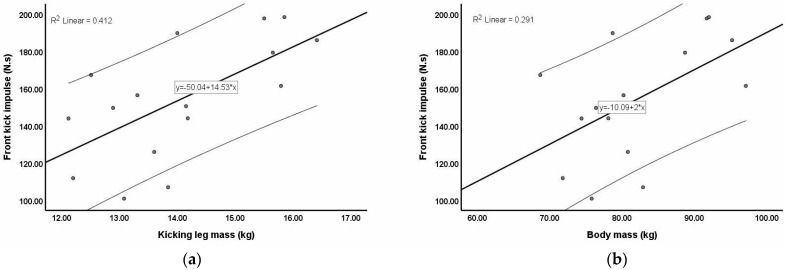
Correlation between the impulse of the front kick and kicking leg mass or body mass: (**a**) kicking leg mass and front kick impulse; (**b**) body mass and front kick impulse.

Figure 3a depicts the relationship between the impulse of the front kick and the kicking leg mass. Figure 3b also shows a positive relationship between the impulse of the front kick and the body mass; however, this relationship was weaker than that between the impulse of the front kick and the kicking leg mass. 

## 4. Discussion

This study explored the relationship between the body mass or the body segment mass and the dynamic forces in combat moves, such as direct punches, palm strikes, elbow strikes, and front kicks. The countermovement jump test, often used for testing acceleration lower limb strength, was also included in this study. Furthermore, our study utilized dual-energy X-ray absorptiometry to provide accurate information on the chosen participants’ body segment mass. That allowed for an analysis correlating the mass of the striking arm and kicking leg with the combat move and the CMJ performance, thereby offering a nuanced understanding of whether knowledge of the mass of individual segments can provide more information about the relationship with the dynamic forces of punches and front kicks than knowledge of the body mass. 

We found a similar relationship between the dynamic forces of the punches and the striking arm mass and a slightly higher relationship between the dynamic forces of the front kick and the kicking leg mass compared to the total body mass. These findings suggested that the chosen body segment masses were not a stronger indicator of the dynamic forces than the participant’s overall body mass. Moreover, in our study, we were also interested in whether there would be different relationships between the individual segment masses or the body mass and the individual dynamic forces such as impulse, peak force, and impact force. The highest relationships were found between the body segment masses or the body mass and the impulse of the front kick, especially for the kicking leg mass with the impulse of the front kick. However, the difference between the explained variability in the front kick impulse using the kicking leg mass and the explained variability in the impulse of the front kick using the body mass was only 12%. Furthermore, the mass of the kicking leg was strongly correlated (the body mass was moderately correlated) with the CMJ impact force, and the front kick impact force and peak force were moderately correlated with the jump height of the CMJ.

### 4.1. Dynamic Forces of the Punches or Front Kicks and CMJ Performance

Comparing the peak force of the direct punch in our study (2501 N) with values from previous studies (3427 N [39], 2600 N [40], 1605 N [41], 1152 N [42], 1659 N [25], 1124 N [43], 2880 N [44]), our finding is in the upper half within the range of previous studies. Similarly, in terms of palm strike, our value of 4210 N was in the upper half of earlier studies (832 N [45], 1593 N [31], 3445 N [46], and 4750 N [44]). Regarding elbow strike, our value of 4677 N fell within the range of values from previous studies (4490 N [44] and 6047 N [46]). Considering the front kick impact force, our value of 3111 N was within the range of previous studies (1620 N [3], 2447 N [33], 2661 N [47], 3600 N [29], and 3691 N [23]) and our value of 6310 N for the front kick peak force was higher than the authors stated in previous studies (5200 N [7], 5551 N [33], and 5604 N [47]). However, the differences between these values in individual studies may be caused by varying levels of participants, stance position, distance from the target, type of target, and often different measuring equipment and the amount or stiffness of the material that covers the striking surface protecting the participants from injury. 

Considering the CMJ performance, our value of 38.43 cm for the jump height was in the range of previous studies (31.1 cm [48], 34.1 cm [49], 35.3 cm [17], 37 cm [50], 39.2 cm [19], 40 cm [51], 44.2 cm [18], and 48.2 cm [13]) that were conducted in groups of military cadets, sport university students, and sub-elite or elite participant level of fighting activities. Regarding peak velocity, our value of 3.1 m/s was slightly higher than in the previous studies (2.55 m/s [48], 2.69 m/s [18], and 3 m/s [35]). The value of the CMJ impact force in our study was 1798 N. Compared with previous studies, it was in the upper half (1190–1235 N [51], 1534–1575 N [19], 1582 N [49], and 1832 N [35]). 

### 4.2. Relationship between Punch or Front Kick Dynamic Forces and Body Mass or Body Segment Mass

Our findings align with the studies suggesting that body mass and composition influence the dynamics of combat techniques. Previous studies have explored the dynamic forces involved in punches and front kicks, highlighting the role of energy production being partially dependent on a subject’s mass in influencing kick dynamics [6,7]. Moreover, we found that the body mass and the body segment mass (striking arm mass or kicking leg mass) showed differential relationships with the peak force, impact force, and impulse achieved during the punch or front kick execution. The relationship between the peak force of the punches or front kicks and the body segment mass was in the range of *r* = 0.11–0.41, while the impulse was in the range of *r* = 0.45–0.64. In previous studies, the authors found that the correlation coefficient between the front kick peak force and the body mass was r = 0.32, and, for the front kick impulse, it was *r* = 0.44 [47]. However, we found that the correlation coefficient between the impact force of the punches or front kicks and the striking arm mass or kicking leg mass or the body mass were in the range of *r* = 0.01–0.16, which does not follow findings in studies in which the correlation coefficients were in the range of *r* = 0.33–0.75 [6,7,52]. Different levels of the participants and gender may explain these contrasting findings [6,7]. For example, in the study by Ramakrishnan et al. [7], novices, sub-elites, women, and men were included in one group. When the authors split this group, the body mass had no significant influence on the kick force in the group of male sub-elite participants. 

### 4.3. Associations between Front Kick Dynamic Forces and CMJ Performance

The CMJ and the front kick are compound movements that rely heavily on the lower limbs’ coordinated action and muscle strength. The CMJ exhibits similar proximal–distal joint coupling to that of the front kick execution [53,54]. Moreover, the CMJ is a reliable indicator of lower body power [55], and the lower body’s power is also important in combat sports performance [19,20]. Therefore, the CMJ is used in conjunction with martial arts performance [18,19,50]. We found a strong relationship between the front kick’s impact force or peak force and the CMJ jump height. The individuals who achieved higher jump heights generated higher impact force and peak force in the front kick. This partially aligns with the study in which the authors found higher CMJ jump heights for Brazilian jiu-jitsu experts than for novices [19]. This finding indicates that the jump height of the CMJ jump is related to the impact and peak forces of the front kick, suggesting that an improvement in the jump height can lead to a higher production of impact force or peak force of the front kick. Regarding the normalized dynamic forces of the front kick using the kicking leg mass compared to using the body mass in relation to CMJ performance, our findings did not suggest that the kicking leg mass is more suitable than normalizing values using the body mass. 

### 4.4. Integration of the Study Findings

Our findings contribute to the current understanding of how the body segment mass influences punches and front kicks. Previous studies have primarily focused on the relationship between the overall body mass and dynamic forces in combat sports [5,6,7,8,9,10]. Our research adds to this body of knowledge by specifically examining the role of the striking arm mass and the kicking leg mass. The significant correlations observed between the kicking leg mass and the front kick impulse suggest that the kicking leg mass can contribute to generating the impulse force during this combat movement. However, many studies mainly focused on peak force and impact force [3,23,29,33,47]. Therefore, based on our findings, we recommend also considering the impulse measured throughout the contact of the striking surface with a fixed target. In practical training, these findings highlight the importance of utilizing the body mass or the kicking leg mass when performing a push kick, in which the fighter aims to exert force on the target area over a longer duration compared to a snap kick. Moreover, the moderate correlations between the front kick impact forces and the jump height in the CMJ underscore the interconnectedness of the lower limb strength and the front kick performance. These insights suggest that training programs focusing on increasing the jump height in the CMJ can also help enhance the impact forces generated when performing a front kick.

### 4.5. Limitations

This study presents a few limitations. Firstly, the sample size is small, comprising only sixteen male military cadets. This limited sample may not provide a comprehensive representation of the broader population engaged in combat sports or military close combat, potentially affecting the generalizability of the findings. Secondly, this study focuses solely on male participants, omitting female personnel, limiting the results’ applicability across genders. Thirdly, this study focused only on military cadets and thus lacked comparison with other groups containing other combat sports, including different skill levels. Therefore, it is possible to generalize the results found only to a selected group of military cadets. This sample was chosen based on their regular involvement in combat training, including punches, kicks, and jumps. Another limitation is the study’s reliance on specific body composition assessment and kinetic measurement techniques (dual-energy X-ray absorptiometry and force plate analysis), which, while accurate, might not be universally accessible for practical application in training environments. Furthermore, this study does not account for the potential influence of the individual’s maximum strength that could affect the dynamic forces of the punches, front kick, and CMJ performance. Lastly, the correlation-based analysis provides insights into associations but does not establish causality.

## 5. Conclusions

This study contributes to understanding how the mass of individual body segments (specifically the striking arm and the kicking leg) correlates with the dynamic forces of combat moves (direct punches, palm strikes, elbow strikes, and front kicks) and CMJ performance in a sample of military cadets. In general, the body segment mass and the body mass showed a higher relationship with the impulse of the punches or the front kick than with impact force or peak force of the punches or the front kick. We found a positive significant correlation between the kicking leg mass and the impulse of the front kicks. However, the difference between the explained variability in the impulse of the front kick using the kicking leg mass and the explained variability in the impulse of the front kick using the body mass was only 12%. Regarding the relationship between the dynamic forces of the punches and the striking arm mass compared to the body mass, no significant relationships were found, and the use of the striking arm mass did not significantly explain the relationship more than the use of the body mass. Finally, our findings did not suggest that normalizing values using the kicking leg mass is more suitable than normalizing values using the body mass in relation to the dynamic forces of the front kick and CMJ performance in a sample of military cadets.

## Figures and Tables

**Table 1:** The performance of punches, front kicks, and countermovement jump and descriptive statistics of body mass and body segment mass.

Dynamic Forces	Direct Punch		Palm Strike		Elbow Strike		Front Kick	
	Mean ± SD	CI Low–Up	Mean ± SD	CI Low–Up	Mean ± SD	CI Low–Up	Mean ± SD	CI Low–Up
Peak force (N)	2501 ± 625	2168–2834	4210 ± 1125	3610–4809	4677 ± 973	4159–5196	6310 ± 1554	5482–7138
Peak force * (N/kg)	474 ± 109	414–534	805 ± 224	681–928	894 ± 196	786–1002	451 ± 108	392–510
Impact time (ms)	17.3 ± 4.2	15.1–19.5	7.9 ± 2.6	6.5–9.2	10 ± 3.7	8.1–12	120 ± 15	8.1–11.6
Impact time * (ms/kg)	3.3 ± 0.8	2.9–3.7	1.5 ± 0.4	1.3–1.7	1.9 ± 0.6	1.6–2.2	8.9 ± 1.2	8.2–9.5
Impulse (N.s)	17.2 ± 3.5	15.3–19.1	14.9 ± 3.6	12.1–18.7	17.6 ± 2.8	16.1–19	154.4 ± 31.5	139–171
Impulse * (N.s/kg)	3.2 ± 0.5	2.96–3.53	2.8 ± 0.6	2.5–3.1	3.3 ± 0.4	3.1–3.6	10.9 ± 1.7	10–12
Impact force (N)	1023 ± 220	906–1141	1968 ± 487	1708–2228	1878 ± 420	1654–2102	3111 ± 735	3719–3502
Impact force * (N/kg)	194.8 ± 41.4	172–218	377 ± 99	322–432	360 ± 88	312–510	223 ± 53	193–252
	Countermovement jump							
	Peak force (N)		Impact force (N)		Peak velocity (m/s)		Jump height (cm)	
Mean ± SD	4531 ± 1051		1798 ± 237		3.10 ± 0.97		38.43 ± 4.72	
CI lower–upper	3971–5091		1672–1924		2.58–3.62		35.92–40.94	
	Body mass (kg)		Striking arm mass (kg)		Kicking leg mass (kg)			
Mean ± SD	82.9 ± 8.1		5.29 ± 6.1		14.1 ± 1.39			
CI lower–upper	77.6–86.7		4.96–5.61		13.32–14.81			

Abbreviations: CI—95% confidence interval, SD—standard deviation. * For the front kicks, values were normalized by the mass of the kicking leg and, for the punches, by the mass of the striking arm.

**Table 2:** Spearman’s correlation coefficients between body segment mass and punches, front kicks, or CMJs.

	Peak Force (N)					Impulse (N.s)				Impact Force (N)					Impact Time (ms)			
	DP	PS	ES	FK	CMJ	DP	PS	ES	FK	DP	PS	ES	FK	CMJ	DP	PS	ES	FK
BM	0.34	0.14	0.20	0.23	0.45	0.55	0.46	0.53	0.52	0.16	−0.09	−0.02	0.01	0.70 *	0.33	0.42	0.27	0.25
SAM	0.28	0.11	0.16	0.31	0.6	0.53	0.45	0.54	0.58	0.14	0.07	0.04	0.19	0.54	0.34	0.43	0.36	0.15
KLM	0.08	0.17	0.04	0.35	0.41	0.46	0.41	0.48	0.64 *	0.08	0.23	0.04	0.14	0.80 *	0.5	0.46	0.30	0.14

Abbreviations: DP—direct punch, PS—palm strike, ES—elbow strike, FK—front kick, CMJ—countermovement jump, BM—body mass, SAM—striking arm mass, KLM—kicking leg mass. All participants in this research were right handed (SAM and KLM are the masses of the right limbs); * *r* ≥ 0.64.

**Table 3:** Spearman’s correlation coefficients between front kick dynamic forces and CMJ performance.

	Countermovement Jump			
	Peak Force (N)	Impact Force (N)	Peak Velocity (m/s)	Jump Height (cm)
FK Peak Force (N)	0.53	0.10	0.63	0.74 *
FK Peak Force * (N/kg)	0.31	−0.32	0.65 *	0.64 *
FK Peak Force ** (N/kg)	0.28	−0.26	0.61	0.63
FK Impulse (N.s)	0.42	0.47	0.16	0.49
FK Impulse * (N.s/kg)	0.25	−0.06	0.24	0.51
FK Impulse ** (N.s/kg)	0.19	0.13	0.09	0.40
FK Impact Force (N)	0.41	−0.10	0.54	0.77 *
FK Impact Force * (N/kg)	0.22	−0.43	0.55	0.63
FK Impact Force ** (N/kg)	0.23	−0.42	0.56	0.58

Abbreviations: FK—front kick; * *r* ≥ 0.64. * The mass of the kicking leg normalized the front kick values. ** The body mass normalized the front kick values.

## Data Availability

The associated dataset for all performed analyses is available at the Open Science Framework [OSF] repository (URL: https://doi.org/10.17605/OSF.IO/KSHBM; DOI:10.17605/OSF.IO/KSHBM), accesed on 1 June 2024.
